# Validated Predictions of Metabolic Energy Consumption for Submaximal Effort Movement

**DOI:** 10.1371/journal.pcbi.1004911

**Published:** 2016-06-01

**Authors:** George A. Tsianos, Lisa N. MacFadden

**Affiliations:** L-3 Applied Technologies, Inc., HEM Division, San Diego, California, United States of America; University of California San Diego, UNITED STATES

## Abstract

Physical performance emerges from complex interactions among many physiological systems that are largely driven by the metabolic energy demanded. Quantifying metabolic demand is an essential step for revealing the many mechanisms of physical performance decrement, but accurate predictive models do not exist. The goal of this study was to investigate if a recently developed model of muscle energetics and force could be extended to reproduce the kinematics, kinetics, and metabolic demand of submaximal effort movement. Upright dynamic knee extension against various levels of ergometer load was simulated. Task energetics were estimated by combining the model of muscle contraction with validated models of lower limb musculotendon paths and segment dynamics. A genetic algorithm was used to compute the muscle excitations that reproduced the movement with the lowest energetic cost, which was determined to be an appropriate criterion for this task. Model predictions of oxygen uptake rate (VO_2_) were well within experimental variability for the range over which the model parameters were confidently known. The model's accurate estimates of metabolic demand make it useful for assessing the likelihood and severity of physical performance decrement for a given task as well as investigating underlying physiologic mechanisms.

## Author Summary

Muscles consume metabolic energy to generate movement. Performing a movement over a long period of time or at a high intensity strains the respiratory and cardiovascular systems that need to replenish the energy reserves in muscle. Furthermore, consuming and replenishing metabolic energy involves biochemical reactions with byproducts that cause muscle fatigue. These biochemical reactions also produce heat that increases body temperature, potentially causing central fatigue. A model of muscle metabolic demand is therefore necessary for predicting and understanding the interaction of these factors that could limit performance, but currently no model exists for arbitrary physical tasks. In this study, we developed a model of metabolic demand by integrating a recently developed and validated model of muscle energetics into a musculoskeletal model. We showed that model predictions for leg exercise over a wide range of intensities were well within the experimental variability reported in the literature. To our knowledge, the muscle energetics model is the first to make valid predictions of metabolic demand at both the muscle and task level. The model is an important step toward understanding and planning around physical performance decrement, which is particularly useful for rehabilitation, competitive sports, and the military.

## Introduction

The ability to achieve and maintain a level of physical performance is important for carrying out activities of daily living and is especially critical for tasks related to the military, competitive sports, and rehabilitation. It is well known that many factors can limit physical performance including the physical demands of the task itself as well as trauma, disease, psychological state, and the environment; however, the underlying mechanisms are poorly understood and current models have limited predictive power. Physical performance emerges from complex interactions among many physiological systems such as cardiovascular, respiratory, and neuromuscular systems. These interactions are largely driven by the metabolic energy demanded; therefore, characterizing metabolic demand across movements is an essential step for revealing the mechanisms of physical performance decrement under the many conditions in which it can occur.

Estimates of metabolic demand for a given muscle or task can indicate susceptibility to muscle fatigue and resulting degradation of performance. Higher rate of muscle energy consumption leads to larger accumulation of chemical byproducts from contraction and metabolism that interfere with active force generation (see [1] for review). High metabolic demand may also exceed the capacity of the cardiorespiratory system to provide the necessary nutrients and to help clear the metabolic and contractile byproducts. A model of metabolic demand would be useful in assessing whether activity can be sustained by the cardiorespiratory system given performance limiting factors such as respiratory muscle fatigue [2, 3], reduced cardiac output [4], reduced partial pressure of oxygen in the inspired air [5] and the presence of gases that may inhibit oxygen absorption [6].

Energy consumption estimates can also help quantify increases in body temperature, which is known to have a strong influence on physical performance. Contractile and metabolic processes are not perfectly efficient and therefore release heat that raises body temperature. Although increases in temperature do not affect muscle function significantly [7], they may be sufficient in hot environments to affect cardiovascular function or to lead to reduced muscle excitation via central mechanisms [8].

Models of metabolic energy consumption for various tasks have been developed in the past, but have limited predictive capabilities. Regression analysis has been used to relate energy consumption to gross characteristics of a task (such as running speed and size of backpack load being carried; [9]), whole body acceleration [10], and joint kinematics and kinetics [11]. Energy consumption has also been related to physiological signals such as electromyographic (EMG) signals [11] and heart rate [12]. Because there are a large number of additional factors with strong, complex, and interactive effects on energetics, regression based models generalize poorly beyond the range of conditions from which they are derived.

Muscle-based models have also been used to predict energetics at the task level [13–15]. These models, however, are limited in their ability to predict muscle activation [16–19], hence energy related to ATP/PCr breakdown. The models are also limited in their ability to predict energy related to ATP/PCr synthesis and together these limitations lead to poor predictions of metabolic energy consumption at the task level (see S4 Appendix).

A muscle energetics model was developed recently and validated for individual muscles as well as for high intensity dynamic knee exercise [17]. However, it is important to validate the model across the full range of task efforts, because muscle mechanics and energetics depend strongly and complexly on activation level [17–19], and because most physiological tasks involve low to moderate levels of muscle activation. For maximal effort tasks, it can be assumed that synergist muscles are activated at similarly high levels [17, 20]. However, predicting energetics of submaximal effort tasks is more challenging because it requires computing the relative activation and load sharing among synergist muscles. The goal of this study was to extend the model to reproduce the kinematics, kinetics, and metabolic energy consumption for dynamic exercise across a wide range of intensities.

## Methods

**Fig 1:**
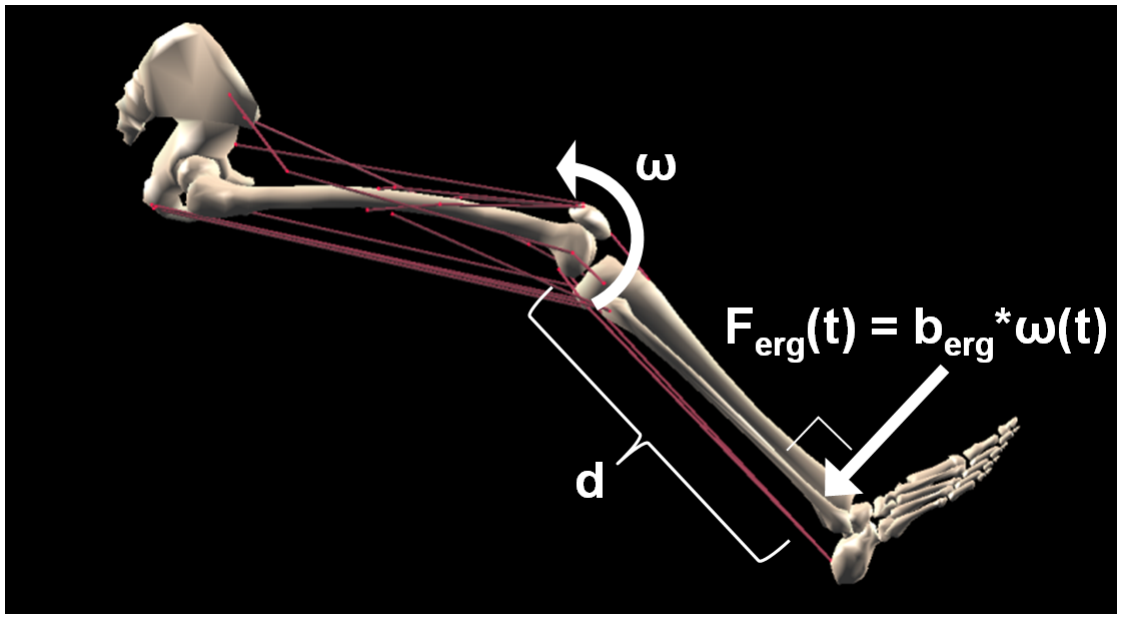
Schematic of dynamic knee extension model. In the experiment modeled [21, 22], subjects performed periodic knee extensions against different loads exerted by a cycle ergometer (see Figure 1 in [21]). A musculoskeletal model of the knee was constructed to model the motion actuated by the muscles crossing the knee as well as the corresponding energy consumed. A model of the cycle ergometer was also included (see text for details). Graphic of musculoskeletal model is a screenshot of the model we constructed in open-source software named MusculoSkeletal Modeling Software (MSMS; [27]).

The following procedure tested whether a validated model of metabolic energy consumption at the muscle level [17] could be extended to predict task energetics of dynamic exercise across various levels of exertion. Dynamic knee extension was modeled because its energetics have been extensively studied and reported in the literature [21–26]. Models of the leg musculoskeletal system, cycle ergometer, and a method for computing task energetics were integrated to simulate the task (Fig 1 shows a schematic of the musculoskeletal plus ergometer model used in this study). The input to the model was the rate of work performed on the cycle ergometer, averaged over one period of the exercise. Given carefully defined parameters that represented the task and musculoskeletal system, the model computed the average rate of metabolic energy consumption of the leg. All of these model parameters were either derived directly from experiments or from independent modeling studies.

### Experimental task modeled

The modeled task is described in Andersen et al. [21] and is briefly summarized here. Subjects were instructed to repeatedly extend one knee against controlled levels of resistance while restrained in an upright sitting posture. They had to extend their knee from 90° to 170° and back in one second, and repeat this motion smoothly and continuously for 8–10 minutes. A heel cup and metal rod assembly connected their leg to the crank arm of a cycle ergometer that resisted knee extension, but allowed knee flexion to occur passively. The task was designed to isolate muscle recruitment to the knee extensor muscles (quadriceps femoris) to facilitate physiological measurements and analysis. These muscles have a large mass so changes in their activity and corresponding energy consumption contributed to most of the change in pulmonary oxygen uptake that was measured [21]. In a separate study [22], blood samples were drawn from the femoral artery and vein to measure oxygen uptake of the quadriceps muscles more directly. Both pulmonary and quadriceps oxygen uptake data were used for validation.

### Musculoskeletal model

A musculoskeletal model was implemented to capture the effects of muscle excitations and external forces such as gravity and ergometer load on the resulting motion and metabolic energy consumption of the leg. The model represented an average young, healthy male with a height of 1.8m and 75kg body mass, which corresponds with the subjects reported in the experimental studies used for validation. With the exception of musculotendon dynamics, all other components of the musculoskeletal model were obtained from a commercially available and validated musculoskeletal model ("Full Body Model"; SIMM, Musculographics, Inc., Santa Rosa, California, United States). To account for leg dynamics, the model includes a set of rigid bodies representing the torso, thigh, shank, and foot segments that link the modeled hip, knee, and ankle joints. Segment dimensions and inertial properties as well as kinematic constraints imposed by the joints are also captured. Limb kinematics are coupled to musculotendon kinematics according to attachment points of musculotendons on the segments and various musculotendon path constraints that account for contact with neighboring soft tissue and bone.

The muscle model described in Tsianos et al. [17] was used to characterize each muscle's force output and rate of metabolic energy consumption in response to neural excitation and musculotendon length. The model accounts separately for the major processes that consume ATP/PCr during a contraction such as active ion transport and cross-bridge cycling. It also accounts for the energy required to replenish ATP/PCr stores (see Figure 11 in [17] and Table 1 in [17] for an overview of the muscle force/energy consumption formulation and for the underlying equations, respectively). The excitation signal in the model represents the common synaptic drive to all motoneurons and ranges between 0 and 1, where 1 corresponds to maximal neural drive. Maximal drive is defined as the level of drive that recruits all motor units at their maximal physiologic firing rate. The excitation signal is transformed into individual motoneuron firing rates according to Henneman's size principle [28, 29]. Muscle fascicle length and velocity is computed from musculotendon length, depending on the relative stiffness of the tendon/aponeurosis and muscle, and muscle mass. The model captures the interactive effects of firing rate, fascicle length, and fascicle velocity on force production and energy consumption. It also accounts for the effects of muscle morphometry and fiber composition.

Morphometric parameters used for this study (see Table 1) were obtained from Delp [30]. The tendon slack length parameter was converted to optimal tendon length (tendon length at maximal isometric force), which is the units of tendon length accepted by the muscle model. Optimal tendon length was defined as 5% shorter than tendon slack length [29]. All muscles in the model were assumed to be composed of equal volumes of slow and fast twitch fiber types, which is consistent with experimental data for the knee extensors [31], the prime movers of the modeled task.

**Table 1:** Modeled muscles and corresponding morphometric parameters. Parameters are identical to those defined in Delp [30]. Fiber type composition of all modeled muscles was set to 50% slow and 50% fast-twitch fibers.

Muscle	Peak isometric force (N)	Optimal muscle fascicle length (cm)	Slack tendon plus aponeurosis length (cm)
*Knee extensors*			
Vastus lateralis	1870	8.4	15.7
Vastus intermedius	1235	8.7	13.6
Vastus medialis	1295	8.9	12.6
Rectus femoris	780	8.4	34.6
*Knee flexors*			
Semimembranosus	1030	8.0	35.9
Biceps femoris long	720	10.9	34.1
Biceps femoris short	400	17.3	10
Semitendinosus	330	20.1	26.2
Gracilis	110	35.2	14.0
Tensor fascia latae	155	9.5	42.5
Sartorius	105	57.9	4.0
Lateral gastrocnemius	490	6.4	38.5
Medial gastrocnemius	1115	4.5	40.8

To implement the musculoskeletal model, the SIMM "Full Body Model" was imported to a modeling environment called MusculoSkeletal Modeling Software [MSMS; 27] that replaced the muscle model from the "Full Body Model" with the validated one described above. Once imported into MSMS, only the right leg was kept and musculoskeletal parameter settings were verified. All joint angles other than the knee were fixed to constant values according to the experiment. Hip flexion angle was fixed to 70° and all other joints were fixed to their neutral anatomical angles (see Fig 1). The MSMS model was then exported to Simulink (The MathWorks, Inc., Natick, Massachusetts, United States), where the differential equations that govern the musculoskeletal system's dynamics were automatically formulated. The ergometer model (see below) was also incorporated into the Simulink model. The aggregate set of differential equations was numerically integrated to determine knee motion and energetics in response to an arbitrary set of muscle excitation signals.

### Ergometer model

A simple ergometer model was constructed based on the ergometer setup and forces recorded in Andersen et al. [21]. In the experiment, subjects were attached to a cycle ergometer through a connecting rod that linked their ankle to the ergometer's crank arm (see Figure 1 in [21]). A resistive load was only applied when the knee was extending. The force acting on the ankle was approximately perpendicular to the long axis of the shank and depended on the rate of knee extension. The force was roughly proportional to the rate of knee extension, so it was defined in the model as the product of knee extension rate and a proportionality constant that depended on the power output of the ergometer:

Ferg(t)={P¯erg2.62*ω(t),ω(t)>00,ω(t)≤0,

where ${\bar{P}}_{erg}$ is the average ergometer power over one period of the task and *ω*(*t*) is knee extension velocity (see S1 Appendix for the derivation of the proportionality constant).

### Computation of task energetics

An optimization algorithm was used to compute the metabolic cost of dynamic knee extension. As explained below, it was assumed that subjects adopted muscle recruitment strategies that minimized metabolic cost, so the optimization algorithm described below was used to predict metabolic cost by computing the muscle excitation signals that minimized the metabolic cost of the dynamic knee extension task.

#### Task definition

To predict the metabolic energy demanded, the task was first carefully defined and then the appropriate neural drive to the musculoskeletal system was computed. The precise definition of the task in the model was determined based on the instructions given to the subjects, the conditions of the task, and observed muscle recruitment strategies. Although experimental subjects were simply instructed to produce periodic knee motion, they likely recruited their muscles in a way that minimized energy consumption. It has been reported that subjects tend to adopt muscle activity that fulfills the task in the most economical way [32, 33], which can help avoid depletion of metabolic energy stores and fatigue (see “Introduction”). It is especially likely that subjects performed dynamic knee extension economically because many repetitions were performed throughout each exercise bout; each bout involved approximately 480 to 600 repetitions (60 extensions/min x 8 to 10 min) with the exception of exhaustive exercise. Moreover, the subjects were encouraged to use their quadriceps muscles mostly, which would prevent them from using the inefficient strategy of co-contraction. The subjects’ inclination toward minimizing energy consumption was captured in the model by defining the task as finding the muscle activity that generated knee kinematics within experimental variability using the least amount of metabolic energy. Traditionally, it has been assumed that simple functions of muscle excitation level, force, and stress are good correlates of metabolic energy, but they differ substantially for many conditions and can lead to substantially different recruitment patterns [17]. In this study, a validated measure of metabolic energy consumption [17] was used to define performance.

**Fig 2:**
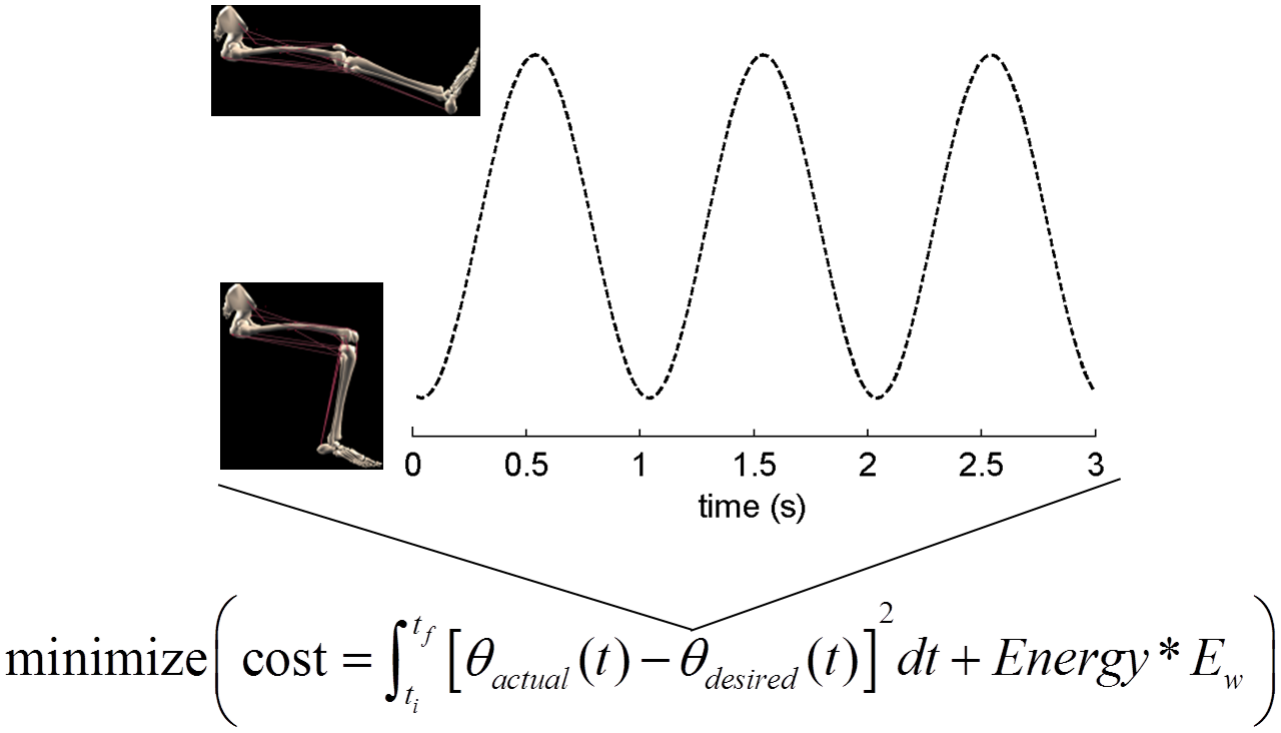
Quantitative definition of the task. The task was defined as minimizing a combination of kinematic error and energetic cost. Kinematic error was a function of the deviation of knee angle from an idealized trajectory (θ_desired_) derived from Andersen et al. [21]. Energetic cost was the rate of metabolic energy consumption of all muscles, averaged over the duration of the exercise. The relative weight of the kinematic and energetic terms (E_w_) was determined such that the optimization algorithm converged reliably to solutions with kinematics within experimental variability at the lowest possible metabolic energy consumption. Graphic of musculoskeletal model is a screenshot of the model we constructed in open-source software named MusculoSkeletal Modeling Software (MSMS; [27]).

Each set of possible muscle excitation signals in the model gave rise to knee motion and a prediction of metabolic energy consumption that together were used to quantify task performance. The inverse of performance, commonly known as cost, was computed as the sum of movement error and metabolic energy consumption scaled by a constant (see equation in Fig 2) and was used to compare performance associated with different muscle recruitment strategies. Movement error was defined as the squared difference of simulated and target knee angle integrated over the entire simulated exercise bout. The square of the deviation from the target angle rather than the absolute value of the deviation was defined as movement error to avoid solutions with large deviations concentrated over short time intervals. This is consistent with subject behavior, which generally involves small deviations from a sinusoidal trajectory throughout the exercise [23]. The target knee angle trajectory was derived from Andersen et al. [21] as a sinusoid bounded by 90° and 170° of extension with a period of 1 second. Rate of metabolic energy consumption was computed by averaging the sum of the rate of energy consumption (energy related to ATP/PCr consumption and synthesis; see [17]) of all muscles over one cycle of the simulated exercise bout. The relative weight of kinematic and energetic error (E_w_) was determined by trial-and-error such that the optimization algorithm (see below) converged reliably to performance that exhibited movement error within experimental variability at minimal metabolic cost. According to Ferguson et al. [23], a typical knee angle trajectory is almost the same as the idealized sinusoidal trajectory depicted in Fig 2. To generate similar trajectories with the model, the allowable movement error (integral of knee angle error squared over one cycle of dynamic knee extension) was set to a small value of 5 deg^2^s per cycle. The precise value of allowable movement error did not have a significant influence on the validation results (see "Results-Sensitivity analysis").

#### Optimization

The model of the knee musculoskeletal system was highly nonlinear and its response to an arbitrary set of muscle excitation signals could not be solved analytically. It was therefore necessary to parameterize muscle excitations and adjust them iteratively to determine which set of muscle excitations generated dynamic knee extension with the least amount of metabolic energy. The signals were represented by periodic rectangular pulses, which can be fully defined by three parameters that account well for muscle activity and performance (see "Results-Exemplary optimization trial") observed experimentally for dynamic knee extension. The period of each pulse was set equal to the period of the simulated task (1 second), while the amplitude, phase (relative to the onset of the simulated task), and duration were tunable parameters. The parameters were constrained to lie within the following ranges:

amplitude: [0,1]
phase: (-period/2, period/2]
duration: [0, period].

The total number of parameters that defined excitation signals to all muscles was 39 (13 muscles * 3 params/muscle). Parameterization schemes with a larger number of free parameters were also tested, but yielded similar results at a higher computational cost.

Optimization was performed to determine the values of parameters defining muscle excitations that reproduce the task. Finding the most energetically economical set of muscle excitations, i.e. muscle recruitment strategies, requires comparison of many sets that satisfy the kinematic criteria of the task. Even for the simple musculoskeletal system of the knee studied here, however, only a subset of possible recruitment strategies could be assessed because the number of possible combinations is prohibitively large. Three parameters defined each of thirteen muscle excitation signals. If each parameter could have one of only five possible values, then the total number of combinations would be (5^3^)^13^, which would be impractical to simulate.

**Fig 3:**
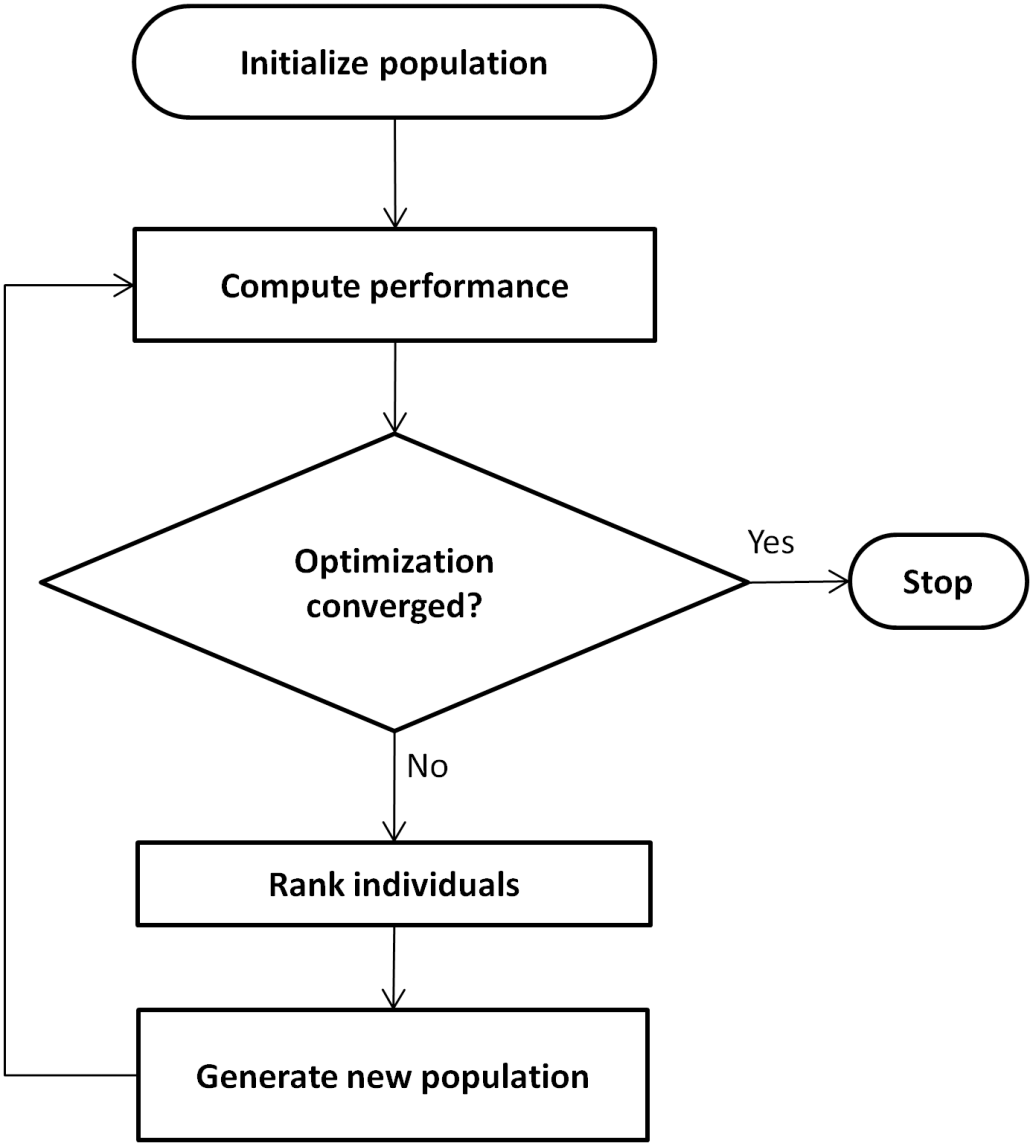
Optimization algorithm. A genetic algorithm was used to compute the muscle excitation parameters that satisfied the performance criteria. See S2 Appendix for a detailed description of each step.

To sample a diverse set of muscle recruitment strategies, global optimization was performed. A genetic algorithm implemented in MATLAB (Global Optimization Toolbox Release 2012b, The MathWorks, Inc., Natick, Massachusetts, United States) was used to randomly initialize a large population of muscle recruitment strategies (i.e. individuals) that were adapted at each iteration of the algorithm (i.e. generation) until performance converged (see Fig 3 for an overview of the algorithm). At each generation, a new population was created whose individuals were generated either by applying random perturbations to individuals from the previous generation (i.e. mutation), taking the weighted average of a pair of individuals (i.e. cross-over), or simply copying individuals to the new generation (refer to S2 Appendix for a detailed description of the algorithm). Ten optimization trials were conducted for each dynamic knee extension load using a different initial population and random number generator seed. The trials converged consistently to kinematic performance within experimental variability and were associated with similarly low levels of metabolic energy consumption. The trial with the lowest metabolic cost was assumed to be most physiological and was used for the validation analysis.

Andersen et al. [21] and Richardson et al. [34] provided evidence that mainly the quadriceps were active during the task while excitation to the other muscles was close to zero. We observed this result in simulation when we used the optimization algorithm to tune excitations of all muscles. To verify that the metabolic energy consumption predicted was near a global optimum, we constrained excitation to all muscles except the quadriceps to zero and reran the optimization. Predictions of metabolic energy consumption were similar, indicating that optimization solutions were close to the global optimum.

### Validation

Predictions of metabolic energy consumption were validated against two separate studies of dynamic knee extension that measured oxygen uptake rate (VO_2_) at the level of the lungs and quadriceps muscles, respectively. The model predicted the steady state energy consumption of the leg in watts. Measuring this directly is highly invasive and complex so there is very little data in the experimental literature. Such data is available for intense dynamic knee extension [25], which has been used previously to validate the model for dynamic exercise at maximum exertion [17]. Typically, the rate of metabolic energy consumption is inferred from VO_2_ measurements. Muscle cells can extract energy from nutrients to drive contraction either with or without oxygen. Nearly all energy gets extracted via a chain of chemical reactions that require oxygen (aerobic catabolism) as long as there is sufficient oxygen present and the rate of ATP/PCr consumption does not exceed the overall reaction rate of aerobic catabolism. The amount of energy extracted per oxygen molecule consumed has been measured and is similar across the different of types of nutrients (carbohydrate, fat, and protein; [35]). For aerobic catabolism, rate of energy consumption in watts can be inferred from VO_2_, measured in typical units of liters O_2_ consumed per minute, using the following conversion factor: 335 [watts]/ [liters O_2_/min] (using 20.1 [kJ]/ [liters O_2_] derived from [36]). If a high amount of anaerobic catabolism is involved, then VO_2_ alone cannot provide a good estimate of metabolic energy consumption and this conversion factor does not apply.

Andersen et al. [21] measured steady state pulmonary VO_2_ (6–8 minutes after exercise onset) from eighteen subjects across several cycle ergometer loads. Dynamic knee extension was modeled for the following ergometer loads: 0, 11, 23.5, 35.5, and 47W. For most subjects, this range of exercise levels involved aerobic catabolism almost exclusively, as arterial lactate did not increase significantly above the resting level [21]. For 47W exercise, arterial lactate levels for some subjects were significantly greater than rest level, but anaerobic relative to aerobic catabolism across subjects appeared to be negligible (also see [37]). Because anaerobic catabolism does not require oxygen, these subjects should theoretically perform the exercise at a lower VO_2_. This is expected to increase variability of VO_2_, but VO_2_ variability for 47W was small and similar to lower exercise loads.

To compare model predictions against the experimental results, the energy consumption rate of the leg muscles was converted to VO_2_. There are other contributors to pulmonary VO_2_ other than skeletal muscles of the moving leg such as the heart and lungs that help supply oxygen to the muscles, and postural muscles that help maintain balance during the exercise. In a separate study, body and leg VO_2_ were measured simultaneously for dynamic knee extension at low to moderate power outputs [24]. Specifically, the ergometer loads tested demanded 18 and 47W of power from the knee extensors, which translates to 0 and 29W of ergometer power given the roughly 18W of power needed to overcome gravity and inertia of the leg [23]. The difference between the measured body and leg VO_2_ was about 0.115 liters of O_2_ per minute for this range of exercise levels and was used to estimate the total energy consumption outside the moving leg for model validation.

In a separate study, Andersen and Saltin [22] reported VO_2_ of the knee extensor muscles for exertion levels ranging from 10W to exhaustion. As in the other validation study, only exercise levels up to about 50W were considered and model output in watts was converted to VO_2_. Experimentally measured VO_2_ also included a component present during rest, whose value was obtained from Krustrup et al. (0.07 liters O_2_/min; [24]) and added to the model prediction of VO_2_ related to the exercise.

### Sensitivity analysis

A sensitivity analysis was performed to assess the robustness of model predictions, given the uncertainty of the most influential parameters. These parameters defined the task and musculoskeletal system. Specifically, hip angle and the lower limit of knee extension range were perturbed by +/-10°. The upper limit of knee extension range was perturbed by +5° and -10°, respectively. The most influential musculoskeletal parameters were simultaneously perturbed within their range of uncertainty (defined in Table 2) to generate the least and most economical musculoskeletal configurations. See S3 Appendix for a detailed explanation and justification for all parameters perturbed.

**Table 2:** Ranges over which musculoskeletal parameters were adjusted for sensitivity analysis. Range limits for each parameter represent one standard deviation above and below the human subject mean. See S3 Appendix for details.

	Vas Lat		Vas Int		Vas Med		Rec Fem	
	lower limit	upper limit	lower limit	upper limit	lower limit	upper limit	lower limit	upper limit
**Optimal muscle fascicle length (cm)**	8.18	11.70	7.90	11.96	7.38	11.98	6.31	8.87
**Slack tendon plus aponeurosis length (cm)**	14.13	17.27	12.24	14.96	11.34	13.86	31.14	38.06
**Fiber composition (% slow-twitch)**	40	60	40	60	40	60	40	60

Sensitivity of energetic predictions was assessed against the variability of pulmonary VO_2_ data from Andersen et al. [21] because the data was collected from a larger and more diverse set of subjects than Andersen and Saltin [22] and is therefore more representative of the variability that could occur from natural variations in these parameters. The two highest ergometer loads (35.5 and 47W) were excluded from the analysis because the contributions to pulmonary VO_2_ from sources besides the leg, such as postural muscles, were not known (see "Methods-Validation").

## Results

Model predictions of metabolic energy consumption across a wide range of dynamic knee extension intensities were within subject variability. This result was robust even when model parameters were varied within their range of possible settings.

### Exemplary optimization trial

**Fig 4:**
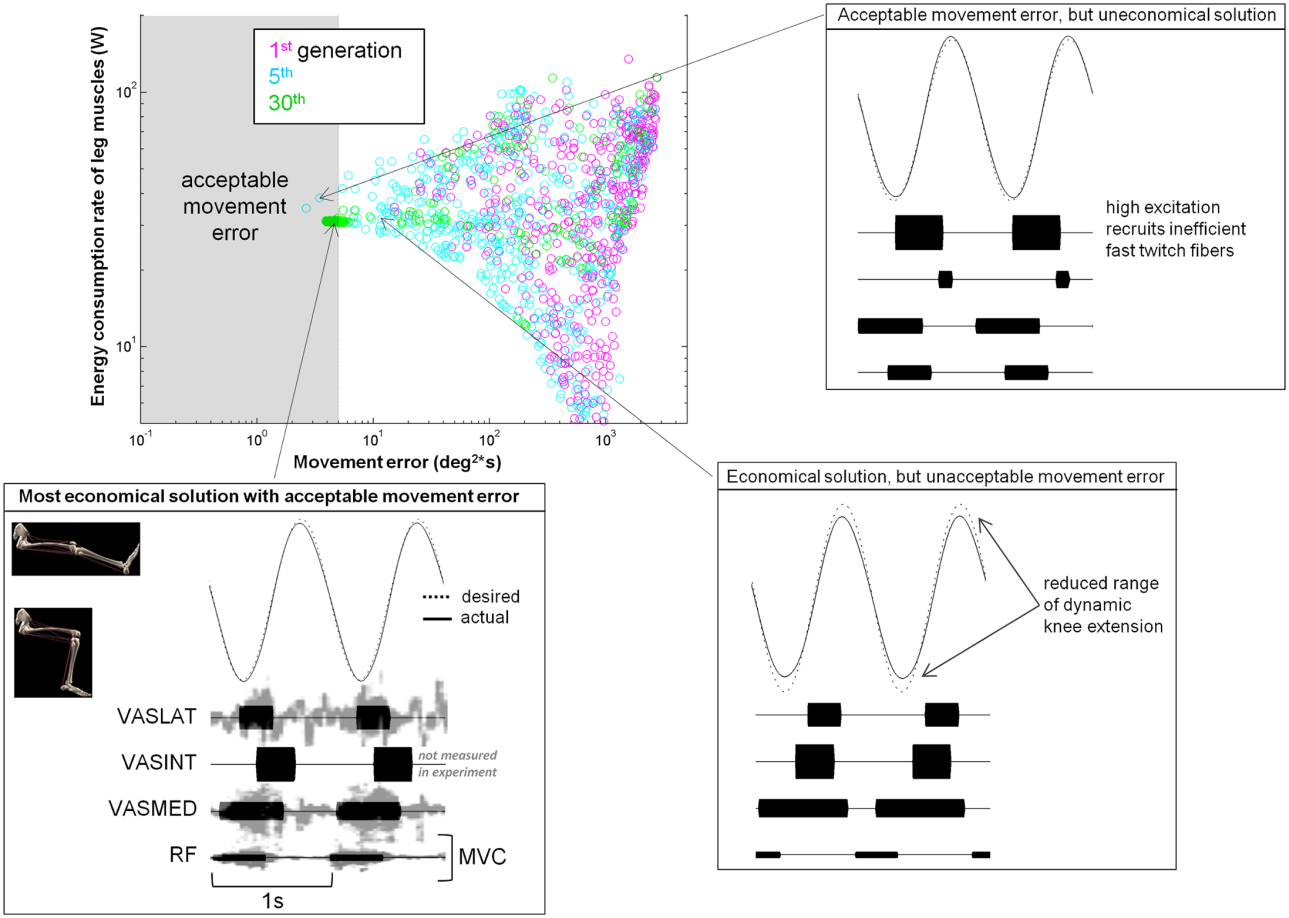
Exemplary optimization trial. The population of solutions discovered by the genetic algorithm is shown for the initial, intermediate, and final generations. Each point in this plot corresponds to one solution, or alternatively, one set of parameters that define the muscle excitation signals. Solutions are plotted in terms of their associated movement error and energy consumption. Solutions within the shaded region exhibited acceptable kinematics. The minimum energy solution that satisfied the kinematic criteria is shown on the bottom left. Experimental muscle activity is shown in gray [21] and is overlaid on model predictions to highlight similarities in timing. An exemplary solution with non-physiological kinematics is shown at the bottom right and non-physiological energetics is at the top right. Graphic of musculoskeletal model is a screenshot of the model we constructed in open-source software named MusculoSkeletal Modeling Software (MSMS; [27]).

An optimization trial and corresponding solution are shown in Fig 4 as an example. Because the initial population of solutions was selected at random, movement error was large and energy consumption rate was highly variable. As optimization progressed, movement error decreased and ultimately converged to acceptable levels at a low energy consumption rate. The solution chosen for the validation analysis was the minimum energy solution with acceptable movement error. Other solutions often exhibited knee angle trajectories that deviated substantially from the specified task or had high energy costs, which could be due to high levels of cocontraction or due to relatively high excitation of one muscle leading to recruitment of less economical fast twitch fibers (see Fig 4).

The optimization algorithm successfully computed muscle excitation signals that reproduced the kinematics of dynamic knee extension in Andersen et al. [21]. As in the experiment, primarily the knee extensor muscles (i.e. the quadriceps) were recruited in the model (also see [34]). The activity of the rest of the muscles was low; it did not contribute significantly to task energetics or kinematic performance. For the trial shown, the timing of modeled and experimentally measured muscle activity via EMG were qualitatively similar. Recruitment patterns that emerged in the model varied substantially depending on the initial population of recruitment patterns used by the genetic algorithm and the sequence of pseudo-random numbers that affected each evolutionary step. Despite having different recruitment patterns, the converged solutions generally met the kinematic criteria at a similarly low energetic cost.

### Validation

**Fig 5:**
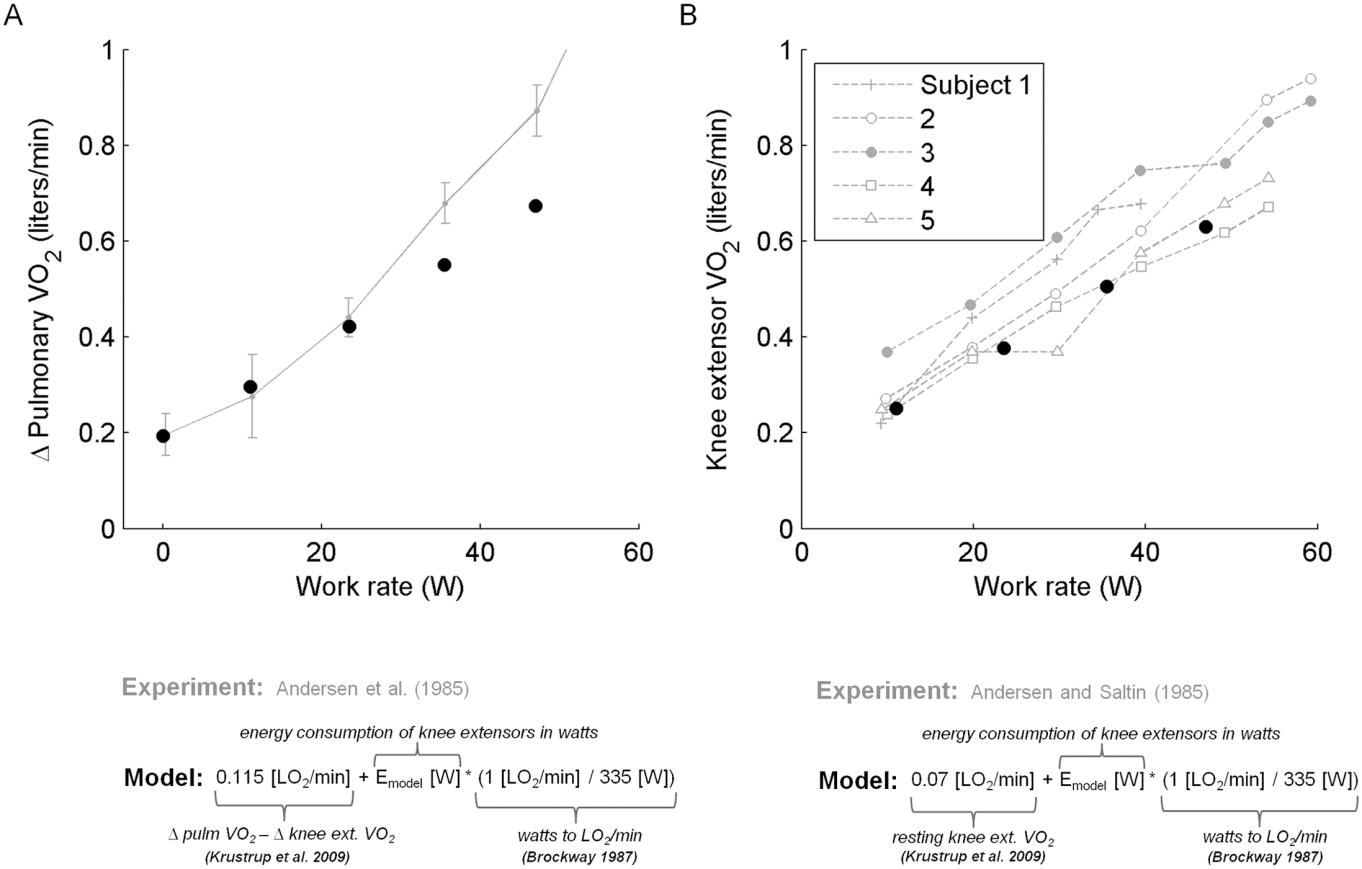
Validation of model predictions. A. Model predictions of energy consumption across dynamic knee extension loads is compared against the rise of pulmonary VO_2_ measured experimentally for eighteen subjects [21]. Δ Pulmonary VO_2_ refers to the increase in steady state rate of oxygen uptake rate by the lungs from rest to exercise. This quantity corresponds to oxygen uptake due to exercise alone (i.e., it does not include the oxygen uptake due to physiological processes contributing to basal oxygen uptake). The model predicts metabolic energy consumption of leg muscles due to exercise (in watts) so to compare it with the Δ Pulmonary VO_2_ data reported, the model output of energy consumption of the leg in watts was converted to liters of oxygen uptake per minute [36] and then added to the oxygen uptake due to exercise from energy sources other than the leg (Δ pulm VO_2_ - Δ leg VO_2_; derived from Krustrup et al. [24]). B. Model predictions are compared against oxygen uptake of the knee extensors measured for five subjects [22]. Knee extensor VO_2_ refers to the steady state rate of oxygen uptake by the knee extensor muscles during exercise. This quantity includes oxygen uptake due to exercise as well as the basal oxygen uptake that is measured at rest. The model predicts metabolic energy consumption due to exercise in watts so to compare it with the knee extensor VO_2_ data reported, the model output of energy consumption of the knee extensors in watts was converted to liters of oxygen uptake per minute [36] and then added to the resting level of knee extensor oxygen uptake obtained from Krustrup et al. [24].

Model predictions of pulmonary VO_2_ were near the center of the experimental range with the exception of the two highest ergometer loads tested (Fig 5; see "Discussion-Validity of model predictions"). Modeled pulmonary VO_2_ increased almost linearly across all work rates while the slope of the experimental pulmonary VO_2_ relation was noticeably higher for higher work rates. Modeled and experimental knee extensor VO_2_ versus work rate were roughly linear. Predictions of knee extensor VO_2_ were within the experimental range across all work rates and were especially similar to measurements from two out of the five subjects in the study.

### Sensitivity analysis

**Fig 6:**
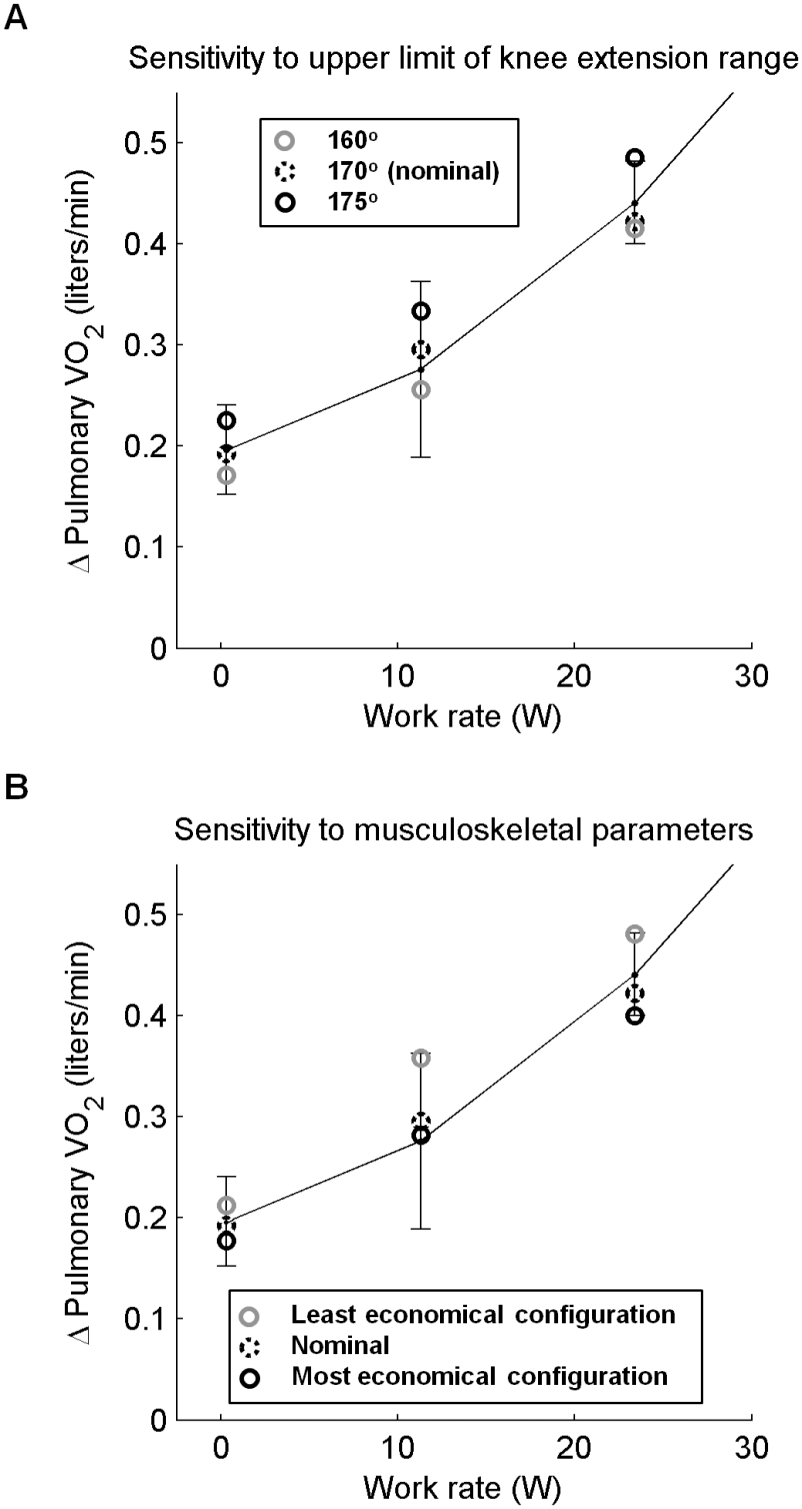
Sensitivity analysis. A. Effect of upper limit of knee extension range on pulmonary VO_2_ predictions. B. Energetic predictions using nominal, least and most economical musculoskeletal configurations. Mean experimental VO_2_ plus/minus one standard deviation is plotted for comparison.

Perturbing the hip angle by +/- 10° led to VO_2_ predictions that were within 3% of nominal, which is well within one standard deviation of experimental variability. Adjusting the lower limit of the knee extension range by +/- 10° resulted in VO_2_ predictions that were on average within 5% of nominal, which is also well within one standard deviation of experimental variability. Changing the upper limit of the knee extension range from 170° to 160° and 175°, respectively, led to deviations that were on average about 20% of nominal VO_2_ predictions. Model predictions in this case remained within one standard deviation of the experimental range and were more sensitive to an increase rather than a decrease in the upper limit of knee extension range (Fig 6a).

The sensitivity of the model's predictions to the acceptable movement error parameter was low. As shown in Fig 4, for example, allowable movement error was chosen conservatively such that the predicted knee angle trajectory was nearly identical to the ideal trajectory (see knee angle trajectory on the bottom left of Fig 4). Even when the accuracy requirement was relaxed to the point where the simulated trajectory deviated substantially from the ideal, as in the bottom right of Fig 4, the metabolic energy predicted remained similar and therefore lay well within experimental variability.

The least and most economical musculoskeletal configurations yielded pulmonary VO_2_ predictions that were within one standard deviation of the experimental variability (Fig 6b). Nominal predictions of pulmonary VO_2_ were closer to those of the least economical configuration. The range of VO_2_ predictions for 11 and 23.5W exercise was about two times larger than 0W exercise.

## Discussion

The goal of this study was to investigate if a validated model of muscle energetics could be used to predict metabolic energy consumption for submaximal effort tasks. Validation and sensitivity results showed that model predictions of metabolic energy consumption for dynamic knee extension across a wide range of workloads agreed well with experimental data for the range over which the model parameters were confidently known. All parameters defining the musculoskeletal system and task were either derived directly from experiments or from independent modeling studies; they were not tweaked to match the model output to experimental results so it is likely that the model is valid across many other tasks and conditions. Accurate predictions of task energetics are important for estimating the load placed on other physiological systems that are involved in carrying out movements. Integrating this model of metabolic demand with models of other physiological systems may shed light into the mechanisms underlying physical performance decrement and help predict performance for a wide range of conditions and subject-specific characteristics.

### Validity of model predictions

Model predictions fell within subject variability for both validation studies, with the exception of predicted pulmonary VO_2_ for the two highest work rates tested. At these high workloads, subjects are likely to increase recruitment of postural muscles to maintain balance, which could lead to the jump in energy consumption observed in the experiment [21]. Postural muscles were not included in the model, therefore, pulmonary VO_2_ predictions did not exhibit such a jump and continued to increase in a nearly linear fashion instead. In fact, the other study conducted by the same group [22] showed that knee extensor VO_2_ rose almost linearly even across these high ergometer loads as opposed to pulmonary VO_2_ (Fig 5). Moreover, model predictions agreed well with these experimental data across all ergometer loads tested.

Varying hip angle did not have a significant effect on model predictions. This can be explained by the fact that when hip angle is altered, out of all knee extensors (the prime movers of this task) only the contractile behavior of the rectus femoris is affected because it is a biarticular muscle that also crosses the hip. The contribution of rectus femoris to the moment underlying dynamic knee extension and corresponding energy consumption is substantially smaller than the other extensor muscles. In the model, rectus femoris has a similar moment arm about the knee with the other knee extensors, but its peak isometric force, hence moment generating capacity was by far the lowest (42–63% of each of the other muscles). Its mass is also substantially lower so its highest possible rate of energy consumption is similarly lower. Furthermore, its fascicle length over the range of motion of the task was relatively small; it was at most 80% of optimal length at the highest hip extension tested while the vasti lengths were 90 to 110% of optimal over the majority of the range. When muscles are operating at lengths below optimal, then the smaller the length, the lower the force generating capacity and metabolic economy (see S3 Appendix and [38]).

Predictions of VO_2_ across tested knee motion ranges were within one standard deviation of the experimental mean. Changing knee extension range changes the knee gravitational and inertial moments of the task, hence the muscle moment and metabolic energy required. Varying the lower limit of knee extension range by +/- 10° had a small effect on VO_2_ predictions because the resulting changes in gravitational and inertial moments had opposite effects on the muscle moment required. For example, reducing the lower limit of knee angle range from 90° extension, where the shank is nearly vertical and gravitational moment is minimal, generates a gravitational moment over the added range of motion that is in the direction of knee extension. This reduces the muscle moment required to decelerate the knee during the terminal phase of knee flexion and to accelerate it during the initial phase of knee extension. At the same time, this reduction of the lower limit of knee extension increases the range of motion, hence the acceleration and inertial moment that the muscles need to overcome. Varying the upper limit of knee extension range resulted in larger effects on VO_2_ predictions because the resulting changes in gravitational and inertial moment both either increased or decreased the necessary muscle moment. Furthermore, increasing the upper limit of knee extension resulted in relatively larger changes in VO_2_ predictions than reducing the upper limit of knee extension partly because this knee angle of 175° is near the anatomical limit of knee extension where passive tension of knee flexors is maximal. Furthermore, the knee extensor lengths decrease over this added range of motion, which as explained in S3 Appendix reduces their metabolic economy. Shorter muscle lengths also reduce the force generating capacity so producing the required level of force for the task requires recruitment of additional fast twitch motor units, which would reduce metabolic economy even further. The large energy consumption and fatigability associated with this high upper limit of knee extension angle along with the possible discomfort makes it unlikely that subjects actually extended their knee this far during the experiment.

**Fig 7:**
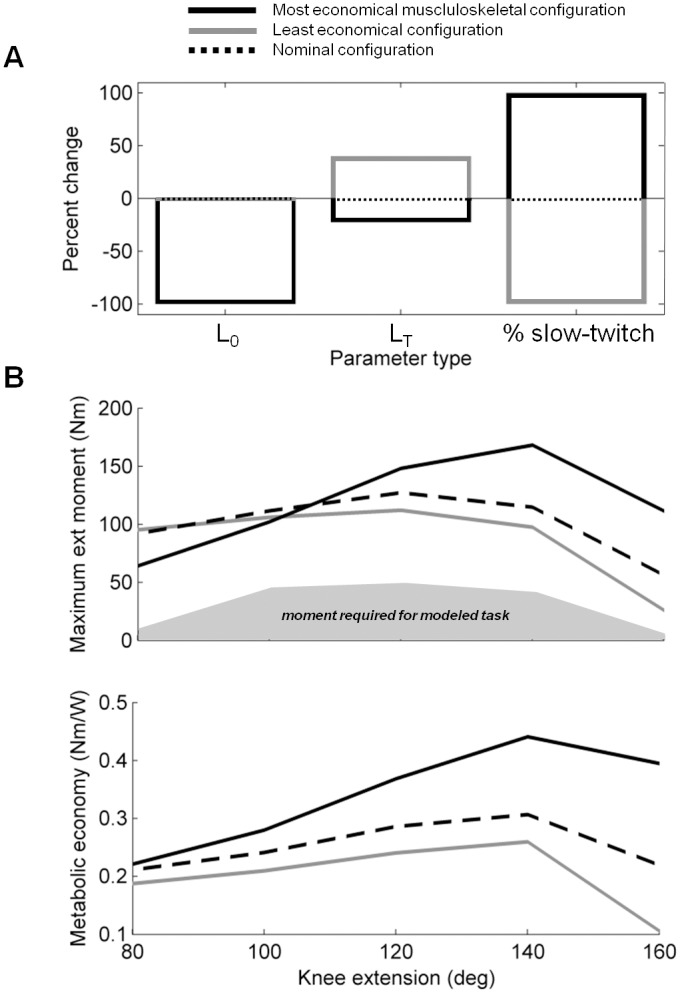
Least and most economical musculoskeletal configurations. A. Musculoskeletal parameter changes that resulted in the least and most economical musculoskeletal configurations. Changes are shown as percentages of the difference between nominal values and one standard deviation above and below the subject mean. B. Maximum moment generating capacity and metabolic economy for maximum muscle excitation is shown for each musculoskeletal configuration. The moment required to perform dynamic knee extension at a 60W ergometer load (i.e. near maximum intensity) is also shown for reference. See S3 Appendix for more a more detailed description of these musculoskeletal configurations.

Pulmonary VO_2_ predictions of the least and most metabolically economical musculoskeletal configurations were within one standard deviation for all work rates tested (see Fig 6b). The range of VO_2_ predictions was substantially higher for 11 and 23.5W than 0W because the least economical configuration consumed substantially more energy at these higher work rates. The relatively low moment generating capacity of the least economical configuration (Fig 7) and smaller percentage of slow twitch fibers required higher activation of the less economical fast twitch fibers to perform 11 and 23.5W exercise. For 0W exercise, the least economical musculoskeletal system could perform the task by recruiting mostly slow twitch fibers like the nominal and most economical musculoskeletal configurations.

Nominal predictions were close to the center of the experimentally measured range of Andersen et al. [21] and variations in model parameters led to reasonable variations of model predictions that remained within one standard deviation of the experimental mean. This is consistent with the fact that the musculoskeletal model was designed to represent an average, young, and healthy male and that experimental measurements were made on a large number of male subjects (18 individuals) with a diverse background of physical activity and a large age range (21–47 years old). The nominal predictions were not as centered on the experimental range of Andersen and Saltin [22]; they generally occupied the lower portion instead. Interestingly, the five healthy men that participated in that experimental study were not as diverse, as they had a narrower age range (21–29 years old) and were all relatively fit. Clearly, the experimental range is substantially larger than the range of predictions made by the model. Experimental measurements outside the range of one standard deviation could reflect subjects with musculoskeletal economy and knee angle trajectories that both bias VO_2_ to either higher or lower levels. Additional parameters such as limb dimensions, inertial properties, fitness and training level also differed across experimental subjects, but were not varied in the model.

The substantial variability of muscle recruitment strategies that emerged in the modeling study has also been observed across experimental subjects [39], although total energy consumption was similar [21]. This is likely a result of having multiple knee extensor muscles with similar moment generating capacity and metabolic economy. For a musculoskeletal system like the one studied here, many different muscle recruitment strategies can lead to similar motion as well as overall metabolic energy consumption. The precise strategy adopted depends on other factors such as the subjects' experience with the task and their perception of the goal. Subjects have their own criteria for performing the task in addition to the explicit instructions given. Some subjects may be more reluctant to change the way they perform a task, in which case the recruitment strategy they end up with would depend strongly on their unique experience. In addition, some muscle recruitment strategies may involve low energy consumption but disproportionate use of muscles could lead to fatigue and associated discomfort rapidly. Subjects are less likely to adopt these muscle recruitment strategies if the duration of the task is long enough to lead to fatigue or if they have a low tolerance for discomfort.

To our knowledge, existing models of muscle energetics [13–15] do not account for important physiological processes that underlie both consumption of metabolic energy to fuel contractions as well as the metabolic energy required to replenish that fuel. As shown in S4 Appendix, these physiological processes have a large influence on metabolic energy consumption and can result in substantial prediction errors if modeled improperly. The model used in this validation effort does not have these limitations and generated predictions within subject variability.

Given the model's accurate predictions of metabolic energy consumption, the underlying assumption that subjects minimized energy while performing the task seems reasonable. In general, people likely minimize energy consumption to conserve fuel for subsequent movements and all other active processes in the body, unless additional factors limit performance. If the goal is to learn the new task quickly, e.g. for survival or competition, minimizing energy would be less of a concern. If peripheral fatigue is a limiting factor, then the probability of fatigue would be minimized instead. Minimizing fatigue may lead to different predictions of muscle recruitment, force, and energetics than simply minimizing energy consumption [40].

### Utility of metabolic demand model in understanding physical performance decrement

Identifying the mechanisms of physical performance decrement requires a better understanding of the interactions among the physiological systems that generate movement. The rate at which the muscular system consumes energy (in the form of ATP) to drive the necessary muscle contractions largely determines these interactions and can be used to quantify them. Metabolic energy consumption is captured at the muscle level by the model in Tsianos et al. [17] and the results presented here provide additional support for its validity, as the model successfully linked the forces necessary to perform the task to the metabolic energy required. The model predicts the rate of energy use required to fuel contractions, which is proportional to the rate of ATP consumption. Model estimates of ATP consumption rate can be used to determine the availability of ATP for subsequent contractions and nutrients for replenishing the ATP used. The model of ATP consumption can also be used to estimate chemical byproducts of contraction, such as inorganic phosphate, that are known to induce muscle fatigue. The type of nutrients and metabolic pathways used is a function of ATP consumption rate [41], so the model can help predict nutrient depletion for different exercises. This also helps predict the extent of glycolytic metabolism, hence H^+^ levels in muscle that can contribute to fatigue. Model predictions of metabolic energy can be used to compute that amount of energy that is not converted to mechanical work, which is dissipated as heat. Estimates of muscle heat output can be used to determine increases in core body temperature that could lead to hyperthermia, hence performance decrement.

ATP consumption determines the amount of nutrients and oxygen that must be supplied by the cardiovascular system. The model can therefore help determine if a given physical task can be supported by the cardiovascular system. Moreover, the amount of oxygen that would need to be absorbed from the environment would also depend on the resistance of the blood vessels supplying the working muscles, which itself is closely related to metabolic demand [42]. Maintaining blood oxygenation also depends on respiratory function that can be affected by environmental factors such as reduced concentration of oxygen in the inspired air or the presence of gases that inhibit oxygen absorption in the blood. Because the oxygen demanded for a task is closely related to the ATP required, the model can be used to assess when lung function limits performance.

Metabolic demand is not monitored in many experimental studies, but can be inferred using this model to assist interpretation of the results and help expand our knowledge of the relationship between metabolic demand and other physiological processes. Even when metabolic demand is monitored, it can be highly inaccurate. Experimentalists typically measure oxygen uptake rate (VO_2_), which can only be used to infer the portion of energy stores consumed that was replenished via oxidative metabolism. Glycolytic metabolism is highly active during intense exercise or in hypoxic conditions [43]; therefore, estimates based on VO_2_ for these situations would be inaccurate. Even if blood lactate is measured, it is difficult to relate it to the extent of glycolytic metabolism because lactate is constantly absorbed by other tissues in the body that ultimately use it as a fuel source or convert it back to glucose [44]. By contrast, the model used here is based on thermodynamic experiments that characterized energy consumption related to contractile processes directly; therefore, its estimates of contractile fuel use do not depend on the type of metabolic pathways involved.

### Conclusion

This paper presents the first valid demonstration of using a muscle contraction model to make accurate predictions of metabolic energy consumption associated with submaximal effort movement. The same modeling approach will likely lead to good predictions across many other tasks and conditions because it accounts for the energetics of individual muscles, it was shown to make valid predictions using only information about the task, and its internal parameters were not tweaked to match experimental results. The results provide additional support for the validity of the muscle energetics model used in this study [17], which is a good starting point for modeling muscle fatigue and nutrient depletion. The modeling approach presented here is useful for relating tasks to the activity of the various physiological systems that are intimately linked with metabolic demand. Using the model and the known functional capacities of the various physiological systems involved, their ability to meet the demands of nonstereotypical or untested tasks and conditions can be investigated. Such integrated analysis would provide insight into the demands placed on each system under a wide range of situations and would therefore help generate testable hypotheses of performance decrement mechanisms.

## Supporting Information

S1 Appendix Ergometer force derivation. (DOCX)

S2 Appendix Optimization algorithm. (DOCX)

S3 Appendix Sensitivity analysis. (DOCX)

S4 Appendix Validity of predictions using other muscle energetics models. (DOCX)
