# Supplementary material for: Validated Predictions of Metabolic Energy Consumption for Submaximal Effort Movement
Source: PLoS Comput Biol. 2016 Jun 1;12(6):e1004911. doi: 10.1371/journal.pcbi.1004911 (PMC4889063; doi:10.1371/journal.pcbi.1004911)
Supplement: S1 Appendix — (DOCX) [file pcbi.1004911.s001.docx]

**S1 Appendix: Ergometer force derivation**

Given the average ergometer power reported, the proportionality constant of the velocity-dependent force was derived as shown below. For this derivation, the knee joint was assumed to be revolute. The point of application of the ergometer force was defined to be at the model's ankle located 43cm away from the knee's center of rotation. The force was assumed to be perpendicular to the long axis of the shank (see Fig. 1 for a schematic).

Instantaneous power: $P_{erg}=F_{erg}(t)*d*\omega_{target}(t)$, where

*F_erg_(t): force applied by the metal rod connecting the ergometer to the ankle*

*d: moment arm of force generated by ergometer = 43cm*

*ω_target_(t): derivative of target knee angle trajectory shown in Fig. 2*

Average power over the interval of one period [t_i_,t_f_]: $\mathop{P}\limits^{\_\_} _{erg}=\frac{\int_{t_{i}}^{t_{f}} F_{erg}(t)*d*\omega_{target}(t)dt}{t_{f}-t_{i}}$

*Force applied only during the extension phase, from t_1_ to t_2_, of the cyclical knee motion*

$$\mathop{P}\limits^{\_\_} _{erg}=\frac{\int_{t_{1}}^{t_{2}} F_{erg}(t)*d*\omega_{target}(t)dt}{t_{f}-t_{i}}$$

*Assuming linear dependence of force on knee extension rate*$\left(F_{erg}(t)=b_{erg}*\omega_{target}(t)\right)$

$$\mathop{P}\limits^{\_\_} _{erg}=\frac{\int_{t_{1}}^{t_{2}} b_{erg}*\omega_{target}(t)*d*\omega_{target}(t)dt}{t_{f}-t_{i}}$$

$$\begin{matrix} & \end{matrix}=\frac{b_{erg}*d\int_{t_{1}}^{t_{2}} [\omega_{target}(t)]^{2}dt}{t_{f}-t_{i}}$$

$$b_{erg}=\frac{\overline{P}_{erg}}{d}\left(\frac{t_{f}-t_{i}}{\int_{t_{1}}^{t_{2}} [\omega_{target}(t)]^{2}dt}\right)$$
