# Supplementary material for: Validated Predictions of Metabolic Energy Consumption for Submaximal Effort Movement
Source: PLoS Comput Biol. 2016 Jun 1;12(6):e1004911. doi: 10.1371/journal.pcbi.1004911 (PMC4889063; doi:10.1371/journal.pcbi.1004911)
Supplement: S2 Appendix — (DOCX) [file pcbi.1004911.s002.docx]

**S2 Appendix: Optimization algorithm**

The optimization algorithm employed in this study was MATLAB's genetic algorithm (Global Optimization Toolbox Release 2012b, The MathWorks, Inc., Natick, Massachusetts, United States). The steps of the optimization algorithm are illustrated in Fig. 3 and a detailed description of their implementation for this study is described below. Unless otherwise stated, the algorithm parameters were set to their default values (see MATLAB 2012b user guide on the Global Optimization Toolbox for more details). The settings of the algorithm that were changed from their default values were configured by trial-and-error to enhance the likelihood and speed of converging to globally optimal performance.

**Initialize population**: 500 individuals were selected at random. Their parameters were chosen from a uniform distribution bounded by parameter constraints defined in "Methods-Computation of task energetics."

**Compute performance**: Each individual was simulated and performance was computed.

**Optimization converged?**: If performance converged, optimization was stopped; otherwise optimization continued. Performance was deemed converged if the best performance out of the population did not improve by more than 1% over five consecutive generations.

**Rank individuals**: Individuals within a population were ranked according to their performance.

**Generate new population**:

- 1. Copy - Two individuals with the best performance were copied to the next generation's population.
  2. Mutation - 30% of the remaining individuals were chosen randomly for mutation, where better ranked individuals were more likely to be chosen. Each selected individual was perturbed in a random direction that satisfied the parameter constraints. The magnitude was initialized to 0.1 and was halved if performance did not improve and doubled if performance improved. The maximum magnitude was set to 0.2.
  3. Cross-over - The remaining individuals (70% of population not "copied" to the next generation) were generated by the "intermediate" cross-over operation. A pair of individuals (i.e. parents), from the previous generation were chosen randomly to generate a new individual. The likelihood that a given individual was chosen as a parent was weighted by "rank." The weighted average of the parents was taken, where the better ranking parents had a larger influence on the generated individuals.
