# Supplementary material for: Validated Predictions of Metabolic Energy Consumption for Submaximal Effort Movement
Source: PLoS Comput Biol. 2016 Jun 1;12(6):e1004911. doi: 10.1371/journal.pcbi.1004911 (PMC4889063; doi:10.1371/journal.pcbi.1004911)
Supplement: S3 Appendix — (DOCX) [file pcbi.1004911.s003.docx]

# S3 Appendix: Sensitivity Analysis

There was some uncertainty in parameter settings related to the task. The inclination of the backrest, which defines hip flexion angle was not reported in Andersen et al. [[1](#_ENREF_1)] and was derived as 70^o^ with respect to the vertical axis from a schematic in Fig. 1 of the article. It was assumed that the modeled hip angle could have deviated from the actual angle by +/- 10^o^ at most. The precise method for measuring knee angle was also not reported. We assumed that the true lower limit of knee extension range may have differed from that reported by +/- 10^o^. Similarly, the true upper limit of knee extension could have been up to 10^o^ less than that reported. It is unlikely that subjects fully extended their knee because this was implied by the instructions to extend to 170^o^. Furthermore, fully extending the knee over the 6-8 minute duration of the task could result in discomfort as well as higher metabolic energy consumption and fatigue susceptibility due to the higher gravitational moment that would have to be overcome (also see "Discussion-Validity of model predictions"). We therefore assumed that all subjects would have not exceeded 175^o^ of extension during the exercise. The effect of these potential deviations in hip and knee angle on the model predictions was tested. The variability of experimental kinematic performance has not been rigorously investigated and therefore such limit could not be determined precisely for this study. The sensitivity of the model's predictions to changes in this parameter was also investigated.

Although experimental and computed estimates of musculoskeletal parameters may have been associated with some error, they likely fell within subject variability, which is consistent with the accurate predictions of metabolic energy consumption (see "Results"). However, it is possible that errors in multiple parameters that would have normally led to poor predictions were compensated by errors in other parameters having the opposite effects on predictions. This possibility was tested by perturbing the parameters and observing the sensitivity of the predictions. Perturbing musculoskeletal parameters individually would not be informative because changes in a parameter of a knee extensor muscle, for example, would be readily compensated by adjustments to neural drive of other extensor muscles with similar actions and metabolic economy. On the other hand, perturbing multiple parameters simultaneously can lead to effects that cancel each other out, as mentioned above, or lead to nonphysiological musculoskeletal models that cannot meet the demands of the task. To avoid these issues and to investigate the worst possible scenarios, knee extensor parameters were perturbed simultaneously such that the metabolic economy was either maximized or minimized while constraining the moment generating capacity of the muscles to be large enough to carry out dynamic knee extension at the maximum intensity that can be sustained by subjects (about 60W on average).

Preliminary experience with manually tuning model parameters showed that model predictions were most sensitive to changes in muscle fascicle length, tendon length, and fiber composition. This is consistent with sensitivity analyses performed on other muscle models (classical Hill-type) showing that muscle force predictions for various simulated tasks are most sensitive to muscle fascicle and tendon length parameters [[2](#_ENREF_2), [3](#_ENREF_3)]. Most and least economical musculoskeletal configurations were created by adjusting all three types of parameters simultaneously. Effects of model parameter changes on task energetics estimates were compared against the experimentally measured standard deviation. To make a fair comparison, parameter perturbation ranges chosen for the sensitivity analysis were also one standard deviation from the subject mean (Table 2). The standard deviation of each muscle's fascicle length was obtained from Ward et al. [[4](#_ENREF_4)], while the standard deviation of slow twitch fiber percentage was obtained from Tirrell et al. [[5](#_ENREF_5)]. To our knowledge, inter-subject variability of tendon slack length of human muscle has only been measured for the gastrocnemius muscle [[6](#_ENREF_6)]. The standard deviation was about 4% of the mean. For this study, we assumed that the standard deviation for knee extensors can be up to 10% of the mean so we allowed the tendon length of each muscle to be perturbed to a value up to 10% greater or lower than nominal.

Pattern search optimization (Global Optimization Toolbox Release 2012b, The MathWorks, Inc., Natick, Massachusetts, United States) was used to find the combination of parameter changes that maximized and minimized metabolic economy, respectively. The optimization was constrained to ensure that the extension moment generating capacity for each joint angle was greater than the moment necessary (computed via inverse dynamics) to perform the task against a 60W load.

The musculoskeletal configurations optimized for low and high metabolic economy are shown in Fig. 7. To create the most economical configuration, the optimization algorithm reduced optimal fascicle lengths of all muscles to their lowest allowable values (see Table 2 for the allowable range of each parameter). Optimal tendon lengths were reduced by 22.5% of the difference between the nominal settings and their lower bounds. Reducing the optimal fascicle and tendon lengths effectively increases the sarcomere length of the muscles. This increased the metabolic economy of the configuration for two main reasons. First, because the muscles operated mostly on the ascending portion of their force-length curve (where formation of some cross-bridges is sterically hindered by overlapping actin filaments), increases in sarcomere length reduce actin overlap and increase the number of cross-bridges for a given calcium concentration in the sarcoplasm. Maintaining a level of calcium requires energy and does not depend on muscle fiber length [[7](#_ENREF_7)]; therefore, for a relatively longer sarcomere length, the same amount of energy would lead to higher force generation. Second, myofilament lattice spacing is smaller at longer lengths, which increases the probability that a given myosin head is close enough to actin binding sites to form a cross-bridge for a given calcium concentration [[see 8](#_ENREF_8)]. Metabolic economy improved further by setting the percentage of slow twitch fibers for all muscles to their maximal allowable value, because slow twitch fibers are known to require substantially less energy than fast twitch muscle to maintain the same force [[9](#_ENREF_9)]. By contrast, to generate the least economical configuration, optimal tendon lengths were increased by 40% of the difference between the nominal settings and their upper bounds, while optimal fascicle lengths were left virtually unchanged. The percentages of slow twitch fibers, in this case, were set to their minimum allowable value.

Maximum knee extension moment of the most economical configuration was smaller than the least economical configuration over small knee extension angles (~80-100^o^), but was substantially greater over larger knee angles (~100-180^o^). The moment generating capacities of both configurations exceeded the moment required at each joint angle to perform the task. Expectedly, metabolic economy of the most economical configuration was higher than the nominal configuration for all joint angles and this difference was larger for larger knee extension angles. By contrast, metabolic economy of the least economical configuration was lower than the nominal one and this difference also grew as a function of knee extension angle. Metabolic economy of the least economical configuration was closer to nominal than the most economical configuration.

## References

1. Andersen P, Adams RP, Sjogaard G, Thorboe A, Saltin B. Dynamic knee extension as model for study of isolated exercising muscle in humans. J Appl Physiol. 1985;59(5):1647-53. Epub 1985/11/01. PubMed PMID: 4066596.

2. Scovil CY, Ronsky JL. Sensitivity of a Hill-based muscle model to perturbations in model parameters. J Biomech. 2006;39:2055-63.

3. Redl C, Margit G, Pandy MG. Sensitivity of muscle force estimates to variations in muscle-tendon properties. Hum Mov Sci. 2007;26:306-19.

4. Ward SR, Eng CM, Smallwood LH, Lieber RL. Are current measurements of lower extremity muscle architecture accurate? Clin Orthop Relat Res. 2009;467(4):1074-82. Epub 2008/10/31. doi: 10.1007/s11999-008-0594-8. PubMed PMID: 18972175; PubMed Central PMCID: PMCPMC2650051.

5. Tirrell TF, Cook MS, Carr JA, Lin E, Ward SR, Lieber RL. Human skeletal muscle biochemical diversity. J Exp Biol. 2012;215(Pt 15):2551-9. Epub 2012/07/13. doi: 10.1242/jeb.069385. PubMed PMID: 22786631; PubMed Central PMCID: PMCPMC3394665.

6. Hoang PD, Herbert RD, Todd G, Gorman RB, Gandevia SC. Passive mechanical properties of human gastrocnemius muscle-tendon units, muscle fascicles and tendons in vivo. J Exp Biol. 2007;210:4159-68.

7. Barclay CJ, Lichtwark GA, Curtin NA. The energetic cost of activation in mouse fast-twitch muscle is the same whether measured using reduced filament overlap or N-benzyl-p-toluenesulphonamide. Acta Physiol. 2008;193:381-91.

8. Tsianos GA, Loeb GE. Muscle physiology and modeling. Scholarpedia. 2013;8(10):12388.

9. Barclay CJ. Mechanical efficiency and fatigue of fast and slow muscles of the mouse. J Physiol. 1996;497(3):781-94.
