# Supplementary material for: Validated Predictions of Metabolic Energy Consumption for Submaximal Effort Movement
Source: PLoS Comput Biol. 2016 Jun 1;12(6):e1004911. doi: 10.1371/journal.pcbi.1004911 (PMC4889063; doi:10.1371/journal.pcbi.1004911)
Supplement: S4 Appendix — (DOCX) [file pcbi.1004911.s004.docx]

# S4 Appendix: Validity of predictions using other muscle energetics models

Muscle models that have been used for estimating task energetics [[1-3](#_ENREF_1)] have two limitations in common that undermine their validity. First, they are based on classical Hill-type muscle models that make poor estimates of muscle activation (i.e. number of active cross-bridges) for submaximal contractions [[4-7](#_ENREF_4)]. This leads to poor predictions of the metabolic energy consumed related to ATP/PCr breakdown. Second, they underestimate the energy expended by metabolic processes that replenish the ATP/PCr consumed (see below). Under aerobic conditions, the energy consumed due to ATP/PCr breakdown is about the same as the energy related to resynthesizing that ATP/PCr for fast twitch muscle fibers and about fifty percent more for slow twitch fibers [[8](#_ENREF_8), [9](#_ENREF_9)].

In addition to having the limitations mentioned above, the Schutte model [[1](#_ENREF_1), [10](#_ENREF_10)] does not account for fiber type effects and is based on data from amphibian muscle, which is known to have different energetic behavior from mammalian muscle [[11-13](#_ENREF_11)]. Predictions of this model were compared against metabolic energy consumption reported in Glaser et al. [[14](#_ENREF_14)] for spinal cord injury patients cycling at 50 rpm against various ergometer loads [[see Figure 5 in 1](#_ENREF_1)]. Two sets of predictions were plotted for the steady state energy consumption of cycling across various work rates. In one case, it was assumed that no ATP/PCr synthesis was occurring and in the other case it was assumed that ATP/PCr synthesis had reached a steady state. For non-fatiguing repetitive tasks, such as this one, the rate of energy consumption related to ATP/PCr synthesis has been shown to reach steady state by the third minute into the task [[15](#_ENREF_15)]. Because the rate of energy consumption in the experiment was averaged over the fourth and fifth minute into the exercise, the second assumption is more realistic and would lead to model estimates that are about one standard deviation above the measured mean. In fact, even in this case the model underestimates the energy due to ATP/PCr synthesis. The model separates the energy related to ATP/PCr breakdown into the component that is converted to mechanical work and the component that is lost as heat. Rather than scaling the energy related to total ATP/PCr breakdown to determine energy ATP/PCr synthesis energy, the model scales only the heat component. Cycling exercise involves mostly positive muscle work, which can be reasonably approximated at 30% of the ATP/PCr breakdown [[11](#_ENREF_11)]. If the model properly accounted for all of the energy related to ATP/PCr synthesis, its estimates would be an additional standard deviation higher above the measured mean. Furthermore, the model did not account for effects of spasticity, contractures and the more probable recruitment of less economical fast twitch fibers when stimulating muscle via surface electrodes. The effects of all of these would decrease efficiency dramatically, yet the model overestimates energy consumption without accounting for them. Model predictions would be even worse for able-bodied subjects, who performed the same task at more than twice the efficiency [[14](#_ENREF_14)].

Like the Schutte model, the energetics model presented by Bhargava et al. [[2](#_ENREF_2)] is also based on amphibian muscle. The validity of the model at the task level was assessed by comparing its predictions of metabolic rate for walking against data from Burdett et al. [[16](#_ENREF_16)]. The model predicted a metabolic rate due to exercising muscles of 4.29 W/kg, which was added to a measure of basal metabolic rate of 1.51 W/kg [[see 17](#_ENREF_17)] to generate a prediction of 5.8 W/kg for total metabolic rate. Energy consumption rate predicted was about 29% higher than the average reported (5.8 vs. 4.5 W/kg); however, the model did not include the energy rate related to ATP/PCr synthesis. Energy consumption rate in the experiment was measured four minutes after the onset of the task so the rate of ATP/PCr synthesis would have reached its maximum steady state value by that time. To properly compare model predictions to data from Burdett et al. [[16](#_ENREF_16)], the contribution of energy related to ATP/PCr synthesis must be added to model predictions. At steady state, the energy rate required for ATP/PCr synthesis is at least as large as the energy rate due to ATP/PCr breakdown (see introductory paragraph of this section). The predicted metabolic rate of 4.29 W/kg corresponds to ATP/PCr breakdown so metabolic rate due to ATP/PCr synthesis is at least as high. Adding the contribution of ATP/PCr synthesis to the total metabolic rate prediction produces a value of at least 10.09 W/kg (5.8 + 4.29), which is about 124% higher than the experimentally reported average.

For the model presented by Umberger et al. [[3](#_ENREF_3)], data collected from isometric human muscle contractions in vivo [[18](#_ENREF_18)] was scaled to estimate energetics of contractions across the physiological range of muscle activation and kinematics. In this experiment, heat output was measured during maximal isometric contractions lasting from five to fifteen seconds. During this short time frame, the underlying ATP/PCr synthesis rate is very little [[8](#_ENREF_8), [19-21](#_ENREF_19)], hence the heat output is almost entirely attributed to ATP/PCr breakdown. To determine metabolic rate under aerobic conditions, Umberger et al. (2003) scaled this energy component related to the contraction by S=1.5, which greatly underestimates the true energy expended due to ATP/PCr synthesis ("S" should be around 2 for fast twitch muscle and 2.5 for slow twitch muscle; see introductory paragraph of this section). Assuming an intermediate scaling factor for typical human muscles of mixed fiber type (S = 2.25), the estimate of dynamic knee extension energy rate at steady state (when metabolism is mostly aerobic) would be 115.5 W/kg, which is about 40% greater than the upper bound reported (87.8 W/kg). Similarly, using the same scaling factor for the locomotion prediction and applying it only to the portion contributed by the modeled muscles (i.e. 4.4-1.2 W/kg), the corrected estimate would be 6 W/kg, which is also about 40% greater than the upper bound reported (4.3 W/kg). The incorrect scaling factor also undermines the validation analysis for cycling [[22](#_ENREF_22)]. Although the value of S used in the study was not reported, it was conservatively assumed to be 1.5. As in the previous analysis, using a reasonable S of 2.25 and applying it only to the energy expenditure of the modeled muscles would result in a whole body energy expenditure that is at least 30% greater than the upper bound reported across all cadences. The model would also incorrectly predict the effects of muscle fiber composition differences on energetics because, as mentioned above, ATP/PCr synthesis in slow twitch muscle has been reported to be substantially less efficient than fast twitch muscle. Properly accounting for this difference would result in more similar overall efficiency of slow and fast twitch fibers and would thus lead to a greatly reduced predicted difference in energetics between the slow and fast twitch model. Although large, the revised prediction errors of this model are actually conservative because like the Schutte model, the Umberger model did not capture the energy cost of ATP/PCr synthesis for the portion of ATP/PCr breakdown that is responsible for performing mechanical work.

The muscle energetics model used here does not have these limitations and makes predictions of task energetics within subject variability. Capturing these aspects of muscle energetics, therefore, seems important in making accurate predictions at the task level. To our knowledge, this is the first muscle model that has been shown to make valid predictions of energetics associated with submaximal effort movement.

## References

1. Schutte LM, Rodgers MM, Zajac FE, Glaser RM. Improving the efficacy of electrical stimulation-induced leg cycle ergometry: an analysis based on a dynamic musculoskeletal model. IEEE Trans Rehabil Eng. 1993;1(2):109-25.

2. Bhargava LJ, Pandy MG, Anderson FC. A phenomenological model for estimating metabolic energy consumption in muscle contraction. J Biomech. 2004;37:81-8.

3. Umberger BR, Gerritsen KGM, Martin PE. A model of human muscle energy expenditure. Comput Methods Biomech Biomed Engin. 2003;6(2):99-111.

4. Perreault EJ, Heckman CJ, Sandercock TG. Hill muscle model errors during movement are greatest within the physiologically relevant range of motor unit firing rates. J Biomech. 2003;36:211-8.

5. Tsianos GA, Rustin C, Loeb GE. Mammalian muscle model for predicting force and energetics during physiological behaviors. IEEE Trans Neural Syst Rehabil Eng. 2012;20(2):117-33. Epub 2011/08/24. doi: 10.1109/tnsre.2011.2162851. PubMed PMID: 21859633.

6. Brown IE, Cheng EJ, Loeb GE. Measured and modeled properties of mammalian skeletal muscle. II. The effects of stimulus frequency on force-length and force-velocity relationships. J Muscle Res Cell Motil. 1999;20:627-43.

7. Brown IE, Loeb GE. Measured and modeled properties of mammalian skeletal muscle: IV. Dynamics of activation and deactivation. J Muscle Res Cell Motil. 2000;21:33-47.

8. Leijendekker WJ, Elzinga G. Metabolic recovery of mouse extensor digitorum longus and soleus muscle. Pflugers Arch. 1990;416:22-7.

9. Barclay CJ, Weber CL. Slow skeletal muscles of the mouse have greater initial efficiency than fast muscles. J Physiol. 2004;559(2):519-33.

10. Schutte LM. Using musculoskeletal models to explore strategies for improving performance in electrical stimulation-induced leg cycle ergometry [Ph.D. Thesis]. Stanford, CA: Stanford University; 1992.

11. Smith NP, Barclay CJ, Loiselle DS. The efficiency of muscle contraction. Prog Biophys Mol Bio. 2005;88:1-58.

12. Kushmerick MJ, Crow M. Chemical energy balance in amphibian and mammalian muscles. Fed Proc. 1982;41(2):163-8.

13. Barclay CJ, Woledge RC, Curtin NA. Energy turnover for Ca^2+^ cycling in skeletal muscle. J Muscle Res Cell Motil. 2007;28(4-5):259-74.

14. Glaser RM, Figoni SF, Collins SR, Rodgers MM, Suryaprasad AG, Gupta SC, et al., editors. Physiologic responses of SCI subjects to electrically induced leg cycle ergometry. IEEE Eng Med Biology Soc, 10th Annu Int Conf; 1988.

15. Gonzalez-Alonso J, Quistorff B, Krustrup P, Bangsbo J, Saltin B. Heat production in human skeletal muscle at the onset of intense dynamic exercise. J Physiol. 2000;524 Pt 2:603-15. Epub 2000/04/15. PubMed PMID: 10766936; PubMed Central PMCID: PMCPMC2269891.

16. Burdett RG, Skrinar GS, Simon SR. Comparison of mechanical work and metabolic energy consumption during normal gait. J Orthop Res. 1983;1:63-72.

17. Anderson FC. A dynamic optimization solution for a complete cycle of normal gait [Ph.D. Thesis]. Austin, TX: University of Texas at Austin; 1999.

18. Bolstad G, Ersland A. Energy metabolism in different human skeletal muscles during voluntary isometric contractions. Eur J Appl Physiol. 1978;38:171-9.

19. Henriksson J, Katz A, Sahlin K. Redox state changes in human skeletal muscle after isometric contraction. J Physiol. 1986;380:441-51.

20. Crowther GJ, Carey MF, Kemper WF, Conley KE. Control of glycolysis in contractin skeletal muscle. I. Turning it on. Am J Physiol Endocrinol Metab. 2002;282:E67-E73.

21. Walsh B, Stary CM, Howlett RA, Kelley KM, Hogan MC. Glycolytic activation at the onset of contractions in isolated Xenopus laevis single myofibres. Exp Physiol. 2008;93(9):1076-84.

22. Umberger BR, Gerritsen KGM, Martin PE. Muscle fiber type effects on energetically optimal cadences in cycling. J Biomech. 2006;39:1472-9.
